# Supplementary material for: Improving spaces for women first responders: A grounded theory on gender equity
Source: PLoS One. 2025 Sep 10;20(9):e0330849. doi: 10.1371/journal.pone.0330849 (PMC12422450; doi:10.1371/journal.pone.0330849)
Supplement: S1 File — (PDF) [file pone.0330849.s001.pdf]

Supplementary File: Interview Guide

Demographics:

1. Can you please tell me what year you were born?
2. What is your gender identity?
    a. Probe: Woman, cis or trans, other, or prefer not to say
3. What is your sexual orientation?
    a. Probe: Straight/heterosexual, lesbian, bisexual, other, or prefer not to say
4. How long have you worked for the service, in years and months?
5. What is your rank, currently?
6. Would you describe your work location as urban, rural, or a mix?
7. What is your relationship status?
    a. Probe: Married, common law, long-term relationship, divorced, widowed, single, or other
8. What level of education do you have?
    a. High school diploma, college diploma/degree, university degree, post-graduate degree
9. How would you categorize your ethnic background?
    a. European, African, Chinese, Indian, etc.
10. And lastly, what is your primary language?
11. How did you hear about the study?

Individual Life course:

1. Can you tell me what initially drew you to work that you do in police/firefighting/paramedicine?
    a. Probe: Did someone inspire you? When did you first consider working in this role?
2. Can you tell a little bit about your process of becoming a police officer/firefighter/paramedic?
    a. Probe: Such as any training or education?
3. Would you be able to tell me how you arrived at/advanced to the position you are in today?
4. Do you think the components of who you are, as discussed in the demographic questions at the beginning, have shaped your experiences as a first responder?
    a. If yes, how?
    a. Probe: your gender, sexual orientation, ethnic background, etc.
5. [If relevant] Can you speak about the circumstances of you leaving the job/taking a leave of absence?
6. [For policer officers:] Can you please tell me about your base training experience at OPC?
    a. How did you feel, as a woman, in this environment? (Supported? Outnumbered?)
    b. How did you feel about the paramilitary structure of the training?

Resiliency and Stress:

7. What factors and personal characteristics do you think are necessary for succeeding in your position?
    a. Probe: Factors like family, social networks, relationship with coworkers, organizational supports.
    a. Probe: Certain personal qualities or attributes like resiliency, self-determination, drive
8. What is your favourite part of your job? What do you like the least?
    a. Are there particular things that provide you happiness or cause you stress?
9. What supports have helped you in this role? What more do you think people could do to support women in your field?
    a. Sources of support such as family and personal networks, colleagues, organizational

Workplace Culture, Diversity and Inclusion:

10. How would you describe your work culture?
    a. Probe: supportive, competitive, team oriented, individualistic, healthy, toxic, etc.
11. Do you feel as though you come to a safe work environment each day?
    a. Please explain
12. Do you think that management is fair in your organization?
    a. Please explain
13. Do you think the women in your organization have the same access to resources as the men do?
    a. Probe: mentorship, opportunities for growth, work space, equipment, etc.

Gender and Professional Roles:

14. Do you think women bring unique skills or perspectives to the work that you do?
    a. If yes, some examples?
15. Do you think women in your profession face unique challenges? If yes, what are they?
    a. Work-life balance
    b. Family
    c. Health (e.g., reproductive health issues)
    d. Institutional challenges (e.g., shift-work, properly fitting equipment, harassment, or discrimination)
16. So, I'm also studying (other two profession), so I'm wondering: How are your professional experiences similar or different to these other two first responder roles we are investigating?
17. Is there anything else you would like to add that we haven't mentioned yet?
    a. Probe: or anything important you would like to reiterate?

Post-Interview Questions/Comments:

- I will be sending you a follow up email with my contact information, if you would like to reach out to me for any reason.

- Would you be interested in receiving a copy of the final thesis once it's completed?

- Would you be willing to share a recruitment poster with some of your colleagues to help spread the word?
- Feel free to stay in touch and reach out anytime.
