# Supplementary material for: Improving spaces for women first responders: A grounded theory on gender equity
Source: PLoS One. 2025 Sep 10;20(9):e0330849. doi: 10.1371/journal.pone.0330849 (PMC12422450; doi:10.1371/journal.pone.0330849)
Supplement: S2 File — (DOCX) [file pone.0330849.s002.docx]

**Key Recommendations for Improving Spaces for Women First Responders**

- Ensure women have access to uniforms and equipment that fit properly, including multiple sets of uniforms.
- Offer on-the-job paid training that provides the skills required for advancements and promotions.
- Support women during and after promotions by providing ongoing training and mentorship.
- Create scheduling approaches that offer workers more opportunities to shape their regular schedules, such as time of day, or days off.
- Establish formal childcare supports with flexible options that address first responders’ specific needs, including non-traditional and overnight hours and extended care options.
- Create legislation which mandates the delivery of equity, diversity, and inclusion (EDI) training to leaders and workers at all professional levels and geographic regions.
