# Supplementary material for: Improving spaces for women first responders: A grounded theory on gender equity
Source: PLoS One. 2025 Sep 10;20(9):e0330849. doi: 10.1371/journal.pone.0330849 (PMC12422450; doi:10.1371/journal.pone.0330849)
Supplement: Attachment — Submitted filename: Response to Reviewers.docx [file pone.0330849.s003.docx]

# Response to Reviewers

PONE-D-24-42534

Improving Spaces for Women First Responders: A grounded theory on gender equity

PLOS ONE

We thank both the editor and the two reviewers for their consideration of our manuscript. We have carefully reflected upon the generous comments provided and have outlined below our responses to the concerns that were raised. We are hopeful that our manuscript will now be considered acceptable for publication in PLOS ONE.

**Journal Requirements:**

When submitting your revision, we need you to address these additional requirements.

1. Please ensure that your manuscript meets PLOS ONE's style requirements, including those for file naming. The PLOS ONE style templates can be found at <https://journals.plos.org/plosone/s/file?id=wjVg/PLOSOne_formatting_sample_main_body.pdf>  and [https://journals.plos.org/plosone/s/file?id=ba62/PLOSOne_formatting_sample_title_authors_affiliations.pdf](https://can01.safelinks.protection.outlook.com/?url=https%3A%2F%2Ftrack.editorialmanager.com%2FCL0%2Fhttps%3A%252F%252Fjournals.plos.org%252Fplosone%252Fs%252Ffile%253Fid%3Dba62%252FPLOSOne_formatting_sample_title_authors_affiliations.pdf%2F1%2F010f0195cc304c4c-745b801e-6f2b-4d57-98b7-554a4ccbdac7-000000%2FpuwQNcQ-AycXjZCfeMF2iI_4tjCcpRYrYCai27VuHgU%3D203&data=05%7C02%7Ckelly.gregory%40mail.utoronto.ca%7Cc6b49eb9383547195cfc08dd6b6e16c5%7C78aac2262f034b4d9037b46d56c55210%7C0%7C0%7C638784843044535038%7CUnknown%7CTWFpbGZsb3d8eyJFbXB0eU1hcGkiOnRydWUsIlYiOiIwLjAuMDAwMCIsIlAiOiJXaW4zMiIsIkFOIjoiTWFpbCIsIldUIjoyfQ%3D%3D%7C0%7C%7C%7C&sdata=eo5UTkm1F7cSYQlwFLNe%2FM80ZRbF2b%2FjKkxbjbtUyIQ%3D&reserved=0).

We have separated the submission into two files, as per the journal requirements: 1) Cover Letter; 2) Title Page and Manuscript. I have also adapted their formatting to align with the journal requirements, and have uploaded the interview guide as Supplementary Material.

2. In the ethics statement in the Methods, you have specified that verbal consent was obtained. Please provide additional details regarding how this consent was documented and witnessed, and state whether this was approved by the IRB.

We have elaborated on this process on line 153, so that the statement now reads as follows:

“Informed consent was obtained through a process approved by the institutional Research Ethics Board where interested volunteers were emailed a letter of information about the research study and an informed consent document. To participate, interested individuals provided a signed copy of the informed consent document in advance of the interview. Participants were also given the option to provide consent orally, in which case, at the beginning of the interview, the lead author would read through the informed consent document and request that the participant respond verbally to the questions in the consent form, while being audio recorded. The lead author archived participant consent on a password-protected computer.”

3. Thank you for stating in your Funding Statement:

“Kelly B. Gregory received financial support to conduct this work from the Social Sciences and Humanities Research Council of Canada, the Joseph-Armand Bombardier Canada Graduate Scholarship (CGS-M), and the Department of National Defence through the Mobilizing Insights in Defence and Security (MINDS) scholarship initiative. Please note there is no grant number associated with these awards.”

Please provide an amended statement that declares *all* the funding or sources of support (whether external or internal to your organization) received during this study, as detailed online in our guide for authors at <http://journals.plos.org/plosone/s/submit-now>.  Please also include the statement “There was no additional external funding received for this study.” in your updated Funding Statement.

Please include your amended Funding Statement within your cover letter. We will change the online submission form on your behalf.

I have shifted this statement to the cover letter, and added the requested statements.

4. We note that you have indicated that there are restrictions to data sharing for this study. For studies involving human research participant data or other sensitive data, we encourage authors to share de-identified or anonymized data. However, when data cannot be publicly shared for ethical reasons, **we allow authors to make their data sets available upon request**. For information on unacceptable data access restrictions, please see <http://journals.plos.org/plosone/s/data-availability#loc-unacceptable-data-access-restrictions>.

Before we proceed with your manuscript, please address the following prompts

a) If there are ethical or legal restrictions on sharing a de-identified data set, please explain them in detail (e.g., **data contain potentially identifying or sensitive patient information**, data are owned by a third-party organization, etc.) and who has imposed them (e.g., a Research Ethics Committee or Institutional Review Board, etc.). Please also provide contact information for a data access committee, ethics committee, or other institutional body to which data requests may be sent.

Since our data are qualitative in nature and our participants are often the only woman working in their professional community, they contain identifying and sensitive information. As a result, making our data publicly available could jeopardize the professional anonymity and safety of our participants.. However, we would consider providing an anonymized data set upon receiving a request, which can be made to our Human Research Ethics Board at researchethics@[XXXXX].

b) If there are no restrictions, please upload the minimal anonymized data set necessary to replicate your study findings to a stable, public repository and provide us with the relevant URLs, DOIs, or accession numbers. Please see <http://www.bmj.com/content/340/bmj.c181.long> for guidelines on how to de-identify and prepare clinical data for publication. For a list of recommended repositories, please see <https://journals.plos.org/plosone/s/recommended-repositories>. You also have the option of uploading the data as Supporting Information files, but we would recommend depositing data directly to a data repository if possible.

Please update your Data Availability statement in the submission form accordingly.

Please note that we have updated our Data Availability statement. For further details, please see our response to question 5.

5. We note that your Data Availability Statement is currently as follows: [All relevant data are within the manuscript and its Supporting Information files.]

Please confirm at this time whether or not your submission contains all raw data required to replicate the results of your study. Authors must share the “minimal data set” for their submission. PLOS defines the minimal data set to consist of the data required to replicate all study findings reported in the article, as well as related metadata and methods (https://journals.plos.org/plosone/s/data-availability#loc-minimal-data-set-definition).

For example, authors should submit the following data:

- The values behind the means, standard deviations and other measures reported;

- The values used to build graphs;

- The points extracted from images for analysis.

Authors do not need to submit their entire data set if only a portion of the data was used in the reported study.

If your submission does not contain these data, please either upload them as Supporting Information files or deposit them to a stable, public repository and provide us with the relevant URLs, DOIs, or accession numbers. For a list of recommended repositories, please see <https://journals.plos.org/plosone/s/recommended-repositories>.

If there are ethical or legal restrictions on sharing a de-identified data set, please explain them in detail (e.g., data contain potentially sensitive information, data are owned by a third-party organization, etc.) and who has imposed them (e.g., an ethics committee). Please also provide contact information for a data access committee, ethics committee, or other institutional body to which data requests may be sent. If data are owned by a third party, please indicate how others may request data access.

In addition to our answer to question 4, the following response has replaced our previous statement on data availability:

*Data cannot be shared publicly because of* **for ethical reasons, as they contain identifying and sensitive information***. Data are available from the University of Waterloo Institutional Data Access / Ethics Committee (contact via* [researchethics@uwaterloo.ca](mailto:researchethics@uwaterloo.ca)*) for researchers who meet the criteria for access to confidential data.*

6. Please ensure that you include a title page within your main document. You should list all authors and all affiliations as per our author instructions and clearly indicate the corresponding author.

We have made these requested edits to the manuscript file.

7. Please update your submission to use the PLOS LaTeX template. The template and more information on our requirements for LaTeX submissions can be found at [http://journals.plos.org/plosone/s/latex](https://can01.safelinks.protection.outlook.com/?url=http%3A%2F%2Ftrack.editorialmanager.com%2FCL0%2Fhttp%3A%252F%252Fjournals.plos.org%252Fplosone%252Fs%252Flatex%2F1%2F010f0195cc304c4c-745b801e-6f2b-4d57-98b7-554a4ccbdac7-000000%2FwlXNyjJmieEmllZgk4OExoFhmczu0vd7zB2MG7i3Er8%3D203&data=05%7C02%7Ckelly.gregory%40mail.utoronto.ca%7Cc6b49eb9383547195cfc08dd6b6e16c5%7C78aac2262f034b4d9037b46d56c55210%7C0%7C0%7C638784843044550180%7CUnknown%7CTWFpbGZsb3d8eyJFbXB0eU1hcGkiOnRydWUsIlYiOiIwLjAuMDAwMCIsIlAiOiJXaW4zMiIsIkFOIjoiTWFpbCIsIldUIjoyfQ%3D%3D%7C0%7C%7C%7C&sdata=edabXpLpZ%2F4AdJ89eRBR2e61jz04ZGzLLxBTOs8coOY%3D&reserved=0).

We have made this requested edit to the manuscript file.

8. Please include your tables as part of your main manuscript and remove the individual files. Please note that supplementary tables (should remain/ be uploaded) as separate "supporting information" files

Please note that we have converted what was originally a table into an in-text list.

**Additional Editor Comments (if provided):**

Reviewer #1: Abstract
Comment 1, Page 4, Line 1: Consider rephrasing “designed”. Emergency response work is not necessarily gendered in its design.

We thank the reviewer for drawing our attention to the wording of the abstract. Our use of the term “designed” was meant to recognize that men have represented the vast majority of individuals who worked as first responders. As a result, the systems put in place to manage and effectively utilize this human resource has been shaped by this circumstance. In referring to ‘systems’, we mean the routine ways of enabling first responders to perform their work, such as through the design of uniforms and equipment, or approaches taken toward scheduling, for instance. By opening with an acknowledgment of this history, we aim to provide context to the reader about the social norms embedded within first response delivery systems, on which present day work continues to operate, because when a dominant characteristic (such as being a man) is so deeply intertwined with the idea or archetype of a social role (such as an emergency responder), it can sometimes appear invisible.

Introduction
Comment 1: Please be consistent with your language, public safety vs. first responder.

We thank the reviewer for the suggestion and have made adjustments to use consistent terminology.

Comment 2: A definition of what a first responder is would be helpful to focus the reader on the cohorts you are specifically commenting on. There is a lot of back and forth in the introduction between FF, PO etc, so it would be help to define the groups you are presenting on.

We thank the reviewer for the suggestion. We have added the following broad definition of “first responders” and clarified the focus of this specific study:

Line 88: “Notably, we take “first response work” to include those professions that respond to and manage medical, safety, or environmental crisis situations in community settings, such as those in the fire service, police service, and paramedicine.”

Line 125: “While there are many first responder roles, including 911 dispatchers and emergency medical technicians, the scope of this project focuses on those most commonly found in the Canadian context: paramedics, firefighters, and police officers.”

Comment 3: It is worth mentioning the increased risk for adverse mental health outcomes among these groups, of which are further elevated among women. EMS and fire, specifically, are noted to have increase depression, anxiety, substance abuse, and even suicide. This speaks volumes when it comes to advocating for more research among these cohorts. Please consider including some of the citations below.

McCann-Pineo M, Keating M, McEvoy T, Schwartz M, Schwartz RM, Washko J, Wuestman E, Berkowitz J. The Female Emergency Medical Services Experience: A Mixed Methods Study. Prehospital Emergency Care. 2024 May 18;28(4):626-34.

Huang G, Chu H, Chen R, Liu D, Banda KJ, O’Brien AP, Jen HJ, Chiang KJ, Chiou JF, Chou KR. Prevalence of depression, anxiety, and stress among first responders for medical emergencies during COVID-19 pandemic: A meta-analysis. Journal of global health. 2022;12.

Stanley IH, Hom MA, Joiner TE. A systematic review of suicidal thoughts and behaviors among police officers, firefighters, EMTs, and paramedics. Clinical psychology review. 2016 Mar 1;44:25-44.

Stanley IH, Boffa JW, Smith LJ, Tran JK, Schmidt NB, Joiner TE, Vujanovic AA. Occupational stress and suicidality among firefighters: Examining the buffering role of distress tolerance. Psychiatry research. 2018 Aug 1;266:90-6.

Lebeaut A, Tran JK, Vujanovic AA. Posttraumatic stress, alcohol use severity, and alcohol use motives among firefighters: The role of anxiety sensitivity. Addictive Behaviors. 2020 Jul 1;106:106353.

We thank the reviewer for the recommendations. We have reviewed and considered them for inclusion.

Comment 4: What was the author’s justification for not including EMT’s? The literature suggest that they experience significant occupational and mental health burdens alongside their paramedic counterparts. There needs to be a firm rationale for their exclusion.

In Canada, when 911 is contacted for a medical emergency, a paramedic is typically dispatched, whereas in the US, it is more typically an EMT (Emergency Medical Technician).We have noted the limits of our focus and suggested that further research should include EMTs as well as other first responders, such as 911 dispatchers:

Line 615: “Lastly, given that we focused on the first responder groups which make up the majority of those who work in the Southern Ontario context, we were unable to investigate the experiences of women who work in first response professions beyond the three previously mentioned. As a result, and in line with the dearth of literature on the subject, future research is required to gain a sense of gender equity in other first response roles, such as Emergency Medical Technicians and 911 dispatchers.”

Comment 5: Has there been any efforts to collect the demographic distribution among the professions? What is the reason for not collecting it? Time, funding? Might be something to comment on.

We thank the reviewer for the reflection. As we described on line 130, many Canadian provinces do not collect gendered demographic data on the first response workforce. Some scholars argue that this is part of a larger and problematic landscape of aggregated provincial and national data which fail to sufficiently provide for analyses of gender, race, and other characteristics.

Methods

Comment 1: Please refer to and utilize the Consolidated Criteria for Reporting Qualitative Research (COREQ) Checklist., There are numerous items that are missing and not included in the main text.

The additional items have been added at various points to the main text.

More detail is need on the recruitment methodology: how many agencies/institutions were provided with the recruitment poster? Besides being active/on leave/retired <3 years, was there any other inclusion/exclusion criteria?

We thank the reviewer for the questions. We have added the requested information, where possible, to line 147. Because our recruitment poster was shared on social media, it remains unclear to us as to how many different agencies and institutions were provided with the recruitment poster.

Who developed the interview guide? Was it reviewed or tested prior to use?

This information has been added on line 175.

Please indicate how long each interview session was.

This information has been added on line 183.

There is no justification for the sample. Was saturation among themes met at 20? How did the authors decide to stop enrollment/interviews?

The following was added on line 191: “At twenty interviews, a repetition of narratives was beginning to occur, therefore, given the richness of the interview data and in recognition of the study’s human resource limitation, enrollment was stopped.”

Were the ZOOM sessions just audio recorded, or video as well? If video was used, please state. Further, if video was used, do you think that responses among those who opted for video/ZOOM may have been biased? Less confidentiality with video, which may have prevented participants from being 100% forthcoming.

Yes, some participants did choose to have their video on while being recorded, while others chose to keep it off - the option was provided upfront to all participants. Further details on this have been added to line 186. For those participants who chose to be video recorded, it was communicated that the audio would be transcribed, and both the audio and video recordings subsequently deleted. Although it is possible that participants who chose to keep their videos on felt differently about sharing their experiences, we will note that the degree of critical or traumatic or challenging experiences that participants shared did not correlate with choosing to be video recorded or not.

Who was responsible for the coding of the transcripts? How do you think the author’s own personal experiences may have influenced the coding? Reflexivity in qualitative research has become increasingly important.

Additional information was added on line 197. It is the lead author’s opinion that their personal experience was more relevant and engaged during the interviewing process, compared to the fairly prescriptive stepwise methodology of coding in constructivist grounded theory (which is detailed on line 199). As such, an additional note has been added to line 169.

Results:

Comment 1: are you able to present the demographic information by profession? This would be helpful for the readers. Further, is there any information on the EMS/fire agency type? Such as EMS agency vs. Fire agency with EMS? This distinction is meaningful and can directly impact participant experiences.

We thank the reviewer for these questions. We have chosen to consolidate the demographic data in an effort to protect the anonymity of participants, as in several circumstances these individuals were the only women in both their profession and their region. Regarding EMS/fire agency type, participants did speak directly to this when their facilities were housed in the same building. However, again, in order to protect the anonymity of participants, we are unable to specify exactly how many were in which arrangement.

Comment 2: The presentation of the four themes is less than to be desired.
Firstly, it appears that there many subthemes within each of the overall themes. For example, ‘Changing Professional Environments’ appears to have subthemes of increases accessibility (uniforms, stair chairs, mobile stretchers, etc), workplace culture (sexism, harassment, women focused policies. This reviewer suggests that you consider reorganizing the presentation of the data to group quotes and ideas by subthemes. It is disjointed as it is currently presented.

We thank the reviewer for sharing their thoughts on this. We feel that the four themes create an important overarching narrative for the paper, which would become lost if we were to present the results by subtheme. We believe the decisions surrounding what is a major theme versus a minor theme can only be ascertained through in-depth engagement with the dataset, and as such, we have respectfully decided to keep the presentation structure unchanged.

Secondly, it may help focus and streamline things if you put all participant quotes within one large table. This would also cut down on the word count.

We thank the reviewer for the suggestion. We feel that presenting the quotes in-line is an important way of keeping participants and their voices in the conversation. We also believe the quotes add richness and humanity to the results, and so prefer to keep them as they are. Should the journal editor prefer to have the quotes presented elsewhere, that may be arranged.

Comment 3: There are numerous occasions where expletives are included in the participant quotes. This review understands “fidelity” of the quotations; however, it is suggested to remove the curse words and replace with something like [expletive].

We thank the reviewer for their thoughts on this. In response, we have made edits to line 370.

Comment 4: Under the ‘Facing resistance” theme, additional participant quotations are need to support the subthemes presented. Specifically, quotes commenting on pregnancy/maternity leave in its relation to career advancements, promotions (particularly being unearned), and social acceptance are needed. These are large paragraphs of text with no supporting quotation.

We thank the reviewer for the feedback. We have added quotes to lines 439 and 455 as requested.

Discussion:

Comment 1: Please include Contursi et al 2018 as an in-text citation.

We thank the reviewer for catching this. It has been corrected.

Comment 2: As mentioned previously, presenting demographic data by profession is necessary to support the claims in your last paragraph of the Discussion.

Unfortunately, we simply cannot disaggregate the demographic data of our participants, because in some circumstances, sharing even their professional role and the fact that they are a woman could enable readers in the field to identify them, given they are the only women in the region in such a position. Further, we feel that presenting the demographics by profession is not necessary to make the claim that this work contributed new knowledge about generational differences, stories of success, or comparisons across the professions.

Comment 3: While this Review appreciates and agrees with the recruitment and retention recommendations presented in Table 1, many of them are anticipated to have low feasibility, particularly among EMS and EMS/Fire agencies with limited funding (i.e rural). At least within the US, the pay discrepancies across the three professions are profound—with EMS receiving a fraction of the salaries of their law and fire counterparts. This generates significant difficulties in instituting any organizational changes that would benefit women directly (i.e scheduling, childcare, etc). Perhaps providing some information on how the Canadian EMS system compares to other major nations would be helpful. Or, maybe provide an example as how the authors envision implementing these recommendations? On paper they are great, but to anyone in operations would see many of these as seemingly impossible to enact.

Although we acknowledge the concern raised by the reviewer, and agree that some of what we have proposed may be difficult to put into practice. We understand that the larger socio-political context and economic constraints might prevent the implementation of these recommendations. And while we believe that not every organization/profession/jurisdiction can implement those, we feel it is still important to list them as they are the needs that were identified by the participants. Further, we feel that our contribution was to identify persistent issues and bring them to the attention of those groups that are in a position to act. Unfortunately, we do not have the organizational insight to determine how feasible some of what we have proposed might be, particularly when, as the reviewer noted, the management and structure of first response professions can vary dramatically across jurisdictions. Instead, our goal was to provide an evidence base underlying the need for greater attention to be paid to women in these professions and to use this evidence to inform recommendations that may be (at the vey least) a starting point for groups in specific regions to begin tackling these ongoing issues.

Secondly, perhaps separating the key recommendations by profession is also needed. Yes, all these recommendations will benefit women in each of the 3 professions, however, there are others that could be specifically tailored for EMS vs Fire vs. Law.

We would like to acknowledge the reviewer’s point, but note that we prefer to keep the recommendations in a more general form to make them accessible to the widest possible audience, as well as to keep the manuscript from becoming too lengthy. As such, these recommendations have been intentionally formatted to be broad and overarching, in order to be relevant across all three professions.

Limitations:

Comment 1: This may no longer be necessary after addressing the comment about saturation, but if saturation was not met this needs to be included as a limitation

We thank the reviewer for the suggestion. The issue of saturation was discussed in the methods section.

Conclusion

There is no conclusion section. Please include.

We thank the reviewer for the suggestion. A conclusion section has been included.

Reviewer #2: This study set out what it aimed to do: to analyze information from women first responders and develop ideas to advance gender equity in the professions. Study significance is supported by the cited literature and using grounded theory to generate new knowledge was effective. The description of the grounded theory process was succinct.

We thank the reviewer for their supportive feedback.

A “major” issue is the abrupt ending of the paper. I wonder if the “Recruitment & Retention” and “Limitations” sections could come earlier in the Discussion section, so there can be a conclusion to wrap up the paper.

We thank the reviewer for this feedback. We have restructured these final sections as suggested.

For clarity, restate the total number of participants in the “Results” section as you begin to describe the population sample. Additionally, defining the term “sexual minority” for your reader would be helpful.

We thank the reviewer for these recommendations – they have been added to lines 211 and 212.

Diving into the topic of first responders who identify as women is important in supporting the increased number of them in these historically masculine professions. I support the publication of this article with the suggested revisions above. Thank you for the opportunity to submit this review.
