# Supplementary material for: Improving spaces for women first responders: A grounded theory on gender equity
Source: PLoS One. 2025 Sep 10;20(9):e0330849. doi: 10.1371/journal.pone.0330849 (PMC12422450; doi:10.1371/journal.pone.0330849)
Supplement: Attachment — Submitted filename: Response_to_Reviewers_auresp_2.docx [file pone.0330849.s004.docx]

# Response to Reviewers

PONE-D-24-42534 R1

Improving Spaces for Women First Responders: A grounded theory on gender equity

PLOS ONE

We thank both the editor and the two reviewers for their consideration of our manuscript. We have carefully reflected upon the generous comments provided and have outlined below our responses to the concerns that were raised. We are hopeful that our manuscript will now be considered acceptable for publication in PLOS ONE.

**Journal Requirements:**

1. If the reviewer comments include a recommendation to cite specific previously published works, please review and evaluate these publications to determine whether they are relevant and should be cited. There is no requirement to cite these works unless the editor has indicated otherwise.

We thank the editor for the recommendations. For further clarity, upon reviewing the suggested articles, we found that McCann-Pineo et al’s 2024 paper to be relevant, and it was added to the literature referenced on line 111. The other articles suggested were not relevant specifically to women’s experiences as first responders, and as a result were not included in the manuscript.

2. Please review your reference list to ensure that it is complete and correct. If you have cited papers that have been retracted, please include the rationale for doing so in the manuscript text, or remove these references and replace them with relevant current references. Any changes to the reference list should be mentioned in the rebuttal letter that accompanies your revised manuscript. If you need to cite a retracted article, indicate the article’s retracted status in the References list and also include a citation and full reference for the retraction notice.

The reference list has been reviewed, and no changes were required.

3. We note that some information in your manuscript text has been anonymized (e.g. ethics committee information on lines 143-144; acknowledgements). Please restore all anonymized information to your manuscript text. If you have any concerns about this request or would like to discuss this further, please contact plosone@plos.org before submitting your revised manuscript files.

We thank the editor for flagging these missing elements. The previously anonymized parts are now included.

**Reviewers' comments:**
Reviewer's Responses to Questions

**Comments to the Author**

1. If the authors have adequately addressed your comments raised in a previous round of review and you feel that this manuscript is now acceptable for publication, you may indicate that here to bypass the “Comments to the Author” section, enter your conflict of interest statement in the “Confidential to Editor” section, and submit your "Accept" recommendation.

**Reviewer #2: All comments have been addressed**

2. Is the manuscript technically sound, and do the data support the conclusions?

The manuscript must describe a technically sound piece of scientific research with data that supports the conclusions. Experiments must have been conducted rigorously, with appropriate controls, replication, and sample sizes. The conclusions must be drawn appropriately based on the data presented.

**Reviewer #2: Yes**

3. Has the statistical analysis been performed appropriately and rigorously?

**Reviewer #2: N/A**

4. Have the authors made all data underlying the findings in their manuscript fully available?

The [PLOS Data policy](https://can01.safelinks.protection.outlook.com/?url=http%3A%2F%2Ftrack.editorialmanager.com%2FCL0%2Fhttp%3A%252F%252Fwww.plosone.org%252Fstatic%252Fpolicies.action%2523sharing%2F1%2F010f01981dac1d6a-69c37dd5-8efc-4c4f-833e-9a5e78f34b86-000000%2FqI5gYhTvvX505PFQ8oSKfn7eHS55FBrTPmwdlJ56BKg%3D220&data=05%7C02%7Ckelly.gregory%40mail.utoronto.ca%7C84f0fd9939b548d57c2d08ddc5fd0b5b%7C78aac2262f034b4d9037b46d56c55210%7C0%7C0%7C638884412845365867%7CUnknown%7CTWFpbGZsb3d8eyJFbXB0eU1hcGkiOnRydWUsIlYiOiIwLjAuMDAwMCIsIlAiOiJXaW4zMiIsIkFOIjoiTWFpbCIsIldUIjoyfQ%3D%3D%7C0%7C%7C%7C&sdata=QhP0eHtsvJjzbj0pQWLs4%2FTH6Q5gZd8nGrSffYgi%2BvM%3D&reserved=0) requires authors to make all data underlying the findings described in their manuscript fully available without restriction, with rare exception (please refer to the Data Availability Statement in the manuscript PDF file). The data should be provided as part of the manuscript or its supporting information, or deposited to a public repository. For example, in addition to summary statistics, the data points behind means, medians and variance measures should be available. If there are restrictions on publicly sharing data—e.g. participant privacy or use of data from a third party—those must be specified.

**Reviewer #2: Yes**

5. Is the manuscript presented in an intelligible fashion and written in standard English?

PLOS ONE does not copyedit accepted manuscripts, so the language in submitted articles must be clear, correct, and unambiguous. Any typographical or grammatical errors should be corrected at revision, so please note any specific errors here.

**Reviewer #2: Yes**

6. Review Comments to the Author

Please use the space provided to explain your answers to the questions above. You may also include additional comments for the author, including concerns about dual publication, research ethics, or publication ethics. (Please upload your review as an attachment if it exceeds 20,000 characters)

Reviewer #2: This revision has increased the impact of this work for publication. Please note some typos in the following lines for correction:
**233-234: "both" does not reference two like things in order. Revise for clarity.
379: Replace "loose" with "lose"
503: Replace "bullshit" with "[expletive]"**

We thank the reviewer for these suggestions.

We have made edits to the statements on 233-234 to add clarity, which now reads as follows:

“Some participants also spoke about increasing accessibility to uniforms and equipment designed for women’s bodies. For example, the paramedic’s stair chair with longer arms and a woman’s MOLLE vest (a tactical vest worn by police officers) are designed to provide more space for breasts in their use, or, in the case of firefighting, smaller oxygen face masks that accommodate a women’s typically more narrow face shape.”

We have also made the suggested edits to lines 379 and 503.

7. PLOS authors have the option to publish the peer review history of their article ([what does this mean?](https://can01.safelinks.protection.outlook.com/?url=https%3A%2F%2Ftrack.editorialmanager.com%2FCL0%2Fhttps%3A%252F%252Fjournals.plos.org%252Fplosone%252Fs%252Feditorial-and-peer-review-process%2523loc-peer-review-history%2F1%2F010f01981dac1d6a-69c37dd5-8efc-4c4f-833e-9a5e78f34b86-000000%2FK0IK6ht_lHlfQKb9h4Jz7U_3156eSONsLF7AWHt3Kg8%3D220&data=05%7C02%7Ckelly.gregory%40mail.utoronto.ca%7C84f0fd9939b548d57c2d08ddc5fd0b5b%7C78aac2262f034b4d9037b46d56c55210%7C0%7C0%7C638884412845382612%7CUnknown%7CTWFpbGZsb3d8eyJFbXB0eU1hcGkiOnRydWUsIlYiOiIwLjAuMDAwMCIsIlAiOiJXaW4zMiIsIkFOIjoiTWFpbCIsIldUIjoyfQ%3D%3D%7C0%7C%7C%7C&sdata=U4%2BSdRK%2FJvtyC1vYp%2Bd5VPfGTrhXeBpwJDHu%2Bc49jA4%3D&reserved=0)). If published, this will include your full peer review and any attached files.

If you choose “no”, your identity will remain anonymous but your review may still be made public.

**Do you want your identity to be public for this peer review?** For information about this choice, including consent withdrawal, please see our [Privacy Policy](https://can01.safelinks.protection.outlook.com/?url=https%3A%2F%2Ftrack.editorialmanager.com%2FCL0%2Fhttps%3A%252F%252Fwww.plos.org%252Fprivacy-policy%2F1%2F010f01981dac1d6a-69c37dd5-8efc-4c4f-833e-9a5e78f34b86-000000%2F7XwYnap59x58oOnXddKGN7-T0KmJRCE60oy36XzQE2o%3D220&data=05%7C02%7Ckelly.gregory%40mail.utoronto.ca%7C84f0fd9939b548d57c2d08ddc5fd0b5b%7C78aac2262f034b4d9037b46d56c55210%7C0%7C0%7C638884412845401730%7CUnknown%7CTWFpbGZsb3d8eyJFbXB0eU1hcGkiOnRydWUsIlYiOiIwLjAuMDAwMCIsIlAiOiJXaW4zMiIsIkFOIjoiTWFpbCIsIldUIjoyfQ%3D%3D%7C0%7C%7C%7C&sdata=38jzal%2F%2BVo7H75fd91%2BhYaci3UArgof4sMF0%2BiGdFR0%3D&reserved=0).

**Reviewer #2: No**
